# On the origin of trisomy 21 Down syndrome

**DOI:** 10.1186/1755-8166-1-21

**Published:** 2008-09-18

**Authors:** Maj A Hultén, Suketu D Patel, Maira Tankimanova, Magnus Westgren, Nikos Papadogiannakis, Anna Maria Jonsson, Erik Iwarsson

**Affiliations:** 1 Warwick Medical School, University of Warwick, UK; 2 Department of Biological Sciences, University of Warwick, UK; 3 Department of Obstetrics and Gynecology, Karolinska Institutet, Sweden; 4 Department of Pathology, Karolinska Institutet, Sweden; 5 Department of Molecular Medicine and Surgery, Karolinska Institutet, Sweden; 6 Department of Pathology, Cardiff University, UK

## Abstract

**Background:**

Down syndrome, characterized by an extra chromosome 21 is the most common genetic cause for congenital malformations and learning disability. It is well known that the extra chromosome 21 most often originates from the mother, the incidence increases with maternal age, there may be aberrant maternal chromosome 21 recombination and there is a higher recurrence in young women. In spite of intensive efforts to understand the underlying reason(s) for these characteristics, the origin still remains unknown. We hypothesize that maternal trisomy 21 ovarian mosaicism might provide the major causative factor.

**Results:**

We used fluorescence in situ hybridization (FISH) with two chromosome 21-specific probes to determine the copy number of chromosome 21 in ovarian cells from eight female foetuses at gestational age 14–22 weeks. All eight phenotypically normal female foetuses were found to be mosaics, containing ovarian cells with an extra chromosome 21. Trisomy 21 occurred with about the same frequency in cells that had entered meiosis as in pre-meiotic and ovarian mesenchymal stroma cells.

**Conclusion:**

We suggest that most normal female foetuses are trisomy 21 ovarian mosaics and the maternal age effect is caused by differential selection of these cells during foetal and postnatal development until ovulation. The exceptional occurrence of high-grade ovarian mosaicism may explain why some women have a child with Down syndrome already at young age as well as the associated increased incidence at subsequent conceptions. We also propose that our findings may explain the aberrant maternal recombination patterns previously found by family linkage analysis.

## Background

Down syndrome (DS) is the most common genetic reason for learning disability and congenital malformations in the human population, occurring with an incidence of around 1/600 newborns. It is now almost half a century since the genetic background was identified, i.e. that most people with DS have an extra small chromosome, some of which are mosaics with only a proportion of trisomy 21 (T21) cells, while a minority have the relevant part of chromosome 21 translocated to another chromosome, leading to this type running in families with a substantially increased risk for parental carriers [1].

**Figure 1:**
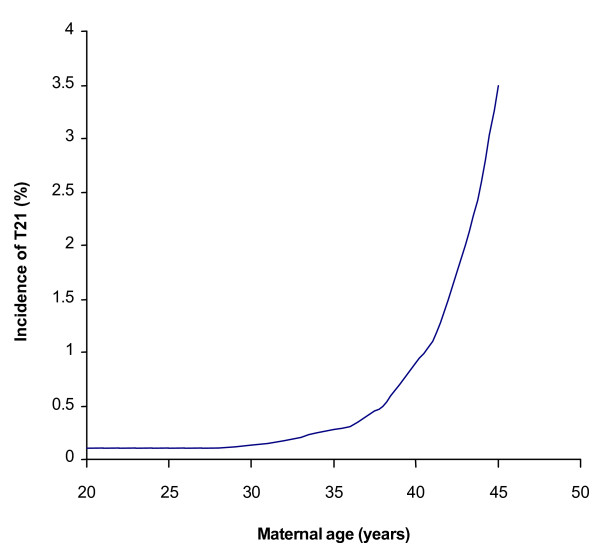
**Birth rate of T21 in relation to maternal age**. The so-called maternal age effect was first recognized by Penrose in 1934, and has since been seen without much variation in different countries around the world.

During the interim five decades much research has been devoted to trace the origin of the common type of DS characterised by an extra free chromosome 21 [2-4]. There is unequivocal evidence from large-scale family linkage studies comparing DNA markers between parents and affected children that the extra chromosome 21 most often originates from the mother, and there may be aberrant maternal recombination along the length of the long arm of chromosome 21. In addition, it is well known that the recurrence is increased for younger mothers and the incidence increases dramatically with maternal age (Figure 1). The underlying biological reasons for this spectrum of clinical and experimental observation are still unknown, constituting outstanding biological enigmas. Most recently it has been suggested that the situation is highly complex, dependent on multifactorial traits [2-4].

**Figure 2:**
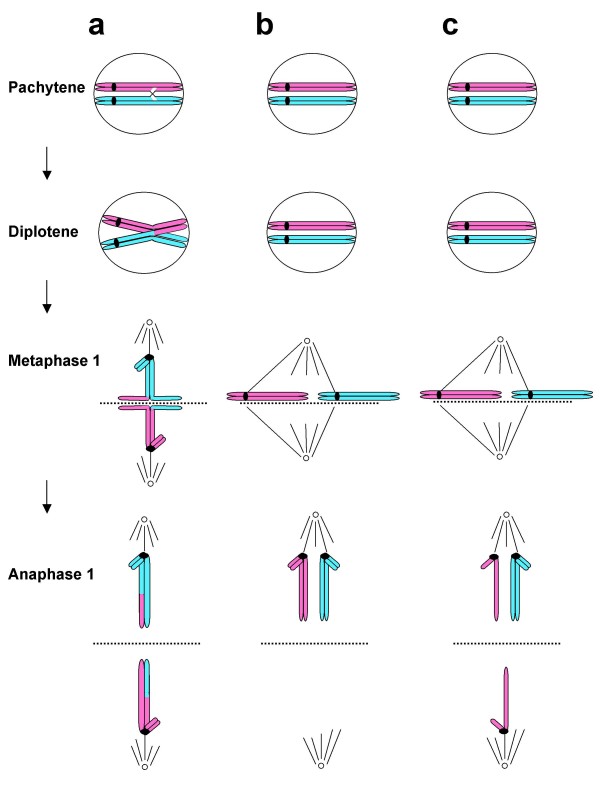
**Cartoon illustrating the different types of meiosis I segregation that may take place in a normal disomy 21 oocyte**. a) Normal chromosome pairing and crossing-over, attachment of the movement centres (kinetochores) at metaphase 1 and separation at anaphase 1. b) Lack of crossing-over and chiasma formation may lead to primary non-disjunction at anaphase 1. c) Lack of a chiasma can also lead to the same type of segregation at anaphase 1 as during mitosis (precocious meiotic disjunction).

**Figure 3:**
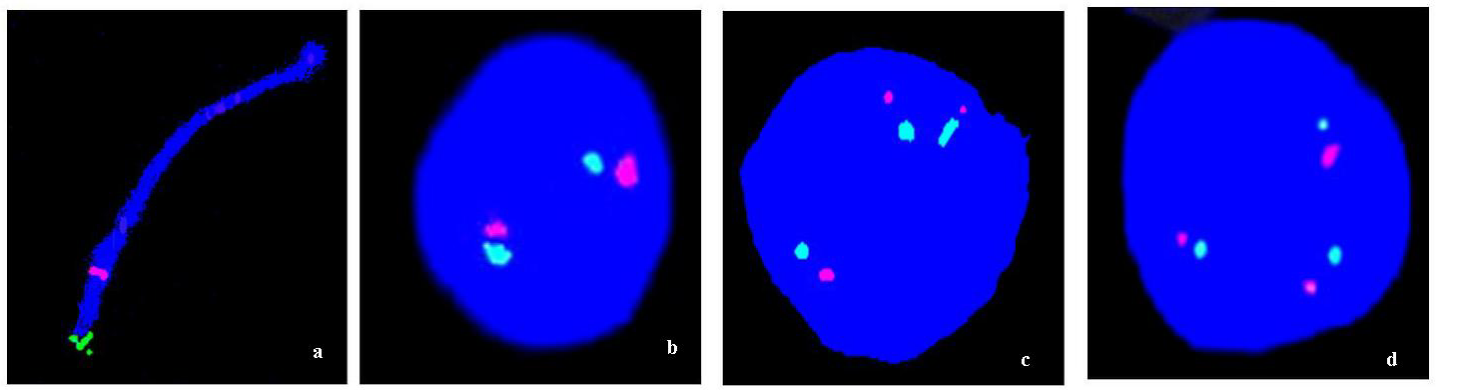
**Examples of FISH results on fetal ovarian cells using two chromosome 21-specific probes**. a) Location of the probes near the end of the long arm of chromosome 21. b) Normal cell nucleus showing two dual chromosome 21-specific signals. c, d) T21 cell nuclei showing three dual chromosome 21-specific signals.

**Figure 4:**
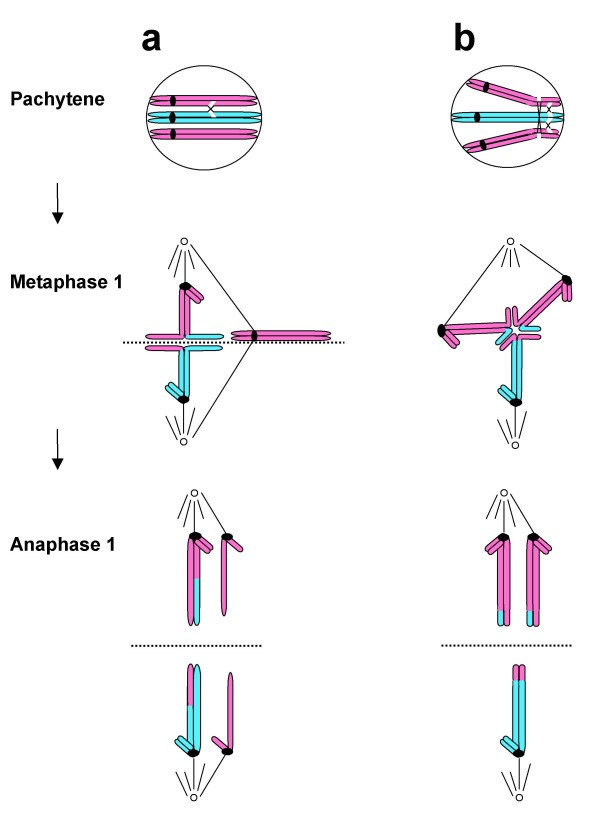
**Cartoon illustrating the different types of secondary non-disjunction that may take place in a T21 oocyte**. a) Formation of a bivalent plus a univalent, where the univalent is undergoing precocious disjunction leading to a chromosome 21 plus a chromatid in each of the daughter cells. b) Formation of a trivalent with chiasmata in an aberrant position, i.e. in this example distally within the long arm. Secondary non-disjunction at anaphase 1 will lead to two chromosomes 21 traveling into one of the daughter cells and one chromosome 21 into the other

Nevertheless it has become generally accepted that the main problem concerns abnormal chromosome behavior of the two chromosomes 21 during the first meiotic oocyte division, so-called primary non-disjunction (Figure 2). We here present data to indicate that the common type of DS in children may in fact result from their mothers themselves being mosaics, carrying a proportion of T21 oocytes in their ovaries (Figure 3). The implication is that the decisive problem is instead the segregation of the three pre-existing chromosomes 21, i.e. obligatory secondary non-disjunction during the first meiotic oocyte division (Figure 4), rather than primary non-disjunction of two chromosomes 21 that has become the generally accepted dogma.

## Materials and methods

All procedures were performed with informed consent and ethical approval from the local ethical committees. Foetal ovarian cells were obtained from eight foetuses at gestational age 14–22 weeks, following termination of pregnancy for social reasons with all the foetuses having a normal phenotypic appearance. Ovaries were removed within a few hours post-mortem and placed in L-15 (Leibovitz) medium (Life Technologies) containing 0.3% bovine serum albumin (Sigma). Pieces of ovaries were frozen at -80°C; thawed cells were prepared for immunoflourescence and fluorescence in situ hybridisation (FISH).

Only a small proportion of each ovary from the initial collection of foetuses [1-4] was used for this study, with the majority divided amongst other experiments, the results of which are described previously [5-7]. In this project we used an antibody against the meiosis-specific protein STAG3 for an initial round of immuno-fluorescence to discriminate between germ cells entering first meiosis prophase in relation to pre-meiotic germ cells and ovarian stroma cells [8]. Parts of the tissue samples from the later collection of foetuses [5-8] were used to prepare direct imprints from the cut surface of the foetal ovary and the remaining material processed by microspreading [9,10].

Microscopy slides for FISH analysis were fixed in methanol: acetic acid then washed in 2X standard saline citrate (SSC) and treated with pepsin (0.1 mg/ml) in 0.01 M HCl for 8 min at 37°C. After additional washing in phosphate-buffered saline (PBS), paraformaldehyde (1%) fixation and dehydration through series of alcohol the slides were left to air-dry at room temperature. Hybridization was performed according to the manufacturers' instructions with two DNA probes positioned near the end of the long arm of chromosome 21 (Cat No: 32-190002, Abbot Molecular Inc, USA and Cytocell, Cat No. LPT21QG/R, Cytocell Technologies Ltd. UK). The DNA probes were mixed and added to the slides followed by denaturation, hybridization and post-hybridization washing. After dehydration slides were mounted in glycerol containing 2.3% DABCO (1, 4-diazabicyclo-(2, 2, 2) octane) as antifade and DAPI (4, 6,-diamino-2-phenyl-indole) 0.5 mg/ml for nuclear counterstaining.

Fluorescent signals were analyzed using a Zeiss Axioskop 2 microscope equipped with a cooled CCD camera (CoolSnap; Photometrics Ltd, USA) controlled by a Power Macintosh computer. Grey scale images were captured, pseudocolored and merged using the SmartCapture 2 software (Digital Scientific Ltd, UK).

The same FISH procedure was applied to preparations of blood lymphocytes obtained from subjects, serving as a control population with respect to the efficacy of the dual FISH probe analysis for appropriate chromosome counts [10]. This included three children with T21 and three children ascertained because of learning disability without any signs of DS, shown to have normal karyotypes by metaphase analysis.

## Results

Using FISH technology with two chromosome 21-specific probes and applying stringent criteria for establishing chromosome 21 copy numbers in foetal ovarian cell nuclei, we detected a proportion of T21 cells in all eight apparently normal female foetuses. Our aim was to analyse at least 1000 foetal ovarian cells in each case and this was possible in all but one of the eight cases. The average incidence of T21 cells was 0.54% with a range of 0.20–0.88% in a total cell population of 12,634 (Table 1). There was no statistically significant difference in incidence between the eight individual cases (SD 0.23; P > 0.05).

**Table 1:** Proportion of T21 cell nuclei in foetal ovaries identified by two chromosome 21-specific probes

		T21 cells (%)		
			
Case No/Id	Gest. Age (wks)	Meiotic prophase germ cells	Pre-meiotic germ and stroma cells	Total (%)
1/C14	17	8/1287 (0.62)	6/637 (0.94)	14/1924 (0.68)
2/C19	18	4/753 (0.53)	2/311 (0.64)	6/1064 (0.56)
3/C17	19	9/1221 (0.74)	7/603 (1.16)	16/1824 (0.88)
4/C11	19	3/392 (0.77)	2/575 (0.35)	5/967 (0.52)
5/F8799	13			6/1578 (0.77)
6/1C	15			2/1048 (0.20)
7/2D	15			6/2029 (0.30)
8/3D	22			13/2200 (0.59)
Average		24/3653 (0.66)	17/2126 (0.80)	68/12634 (0.54)

Note: In cases 1–4 the proportion of germ cells entering meiotic prophase was pre-identified by the meiosis-specific protein STAG3 before the FISH analysis of chromosome 21 copy numbers.

In these T21 figures we have only included those showing three clear double signals in comparison to the normal two. This is illustrated in Figure 3. The left image shows the position of the two fluorescence probes near the end of the long arm of chromosome 21 (Figure 3a). An example of a normal cell nucleus containing two double chromosome 21-specific fluorescence signals (one red and the other yellow-greenish) is shown in Figure 3b. Cell nuclei, each containing three double signals, thus interpreted to have three copies of chromosome 21 and labelled as representing T21 cell nuclei, are illustrated in Figure 3c, d.

Pre-staining with an antibody against the meiosis-specific protein STAG3 performed in cases 1–4 allowed differentiation between germ cells entering meiosis in relation to pre-meiotic germ cells and ovarian stroma cells. The proportion of T21 cells identified by the STAG3 antibody/nuclear size as meiotic germ cells was on average 0.41% with a range of 0.31–0.49%. There was no statistically significant difference between samples in this respect (SD 0.17; P > 0.05).

Using the same criteria as for FISH analysis of foetal ovaries we did not find any indication of a similar type of mosaicism as regards *in vitro *cultured blood lymphocytes from three children with T21 and three children with normal karyotypes by traditional metaphase analysis. Thus, in a total of 12,320 cells analyzed we did not detect any cell nucleus showing extra dual chromosome 21 signals.

## Discussion

### Most women may be T21 ovarian mosaics

We have found that all eight ovaries obtained from foetuses, where termination of pregnancy has been performed for social reasons at gestational age 14–22 weeks, contain a proportion of cells with an extra chromosome 21. In other words these apparently normal female foetuses are ovarian mosaics with trisomy 21 in 0.20–0.88% of cells. It is tempting to conclude that this type of ovarian mosaicism may be a general constitutional characteristic of our species, underlying the common occurrence of T21 conceptions and caused by the obligatory first meiotic segregation at ovulation of two of the three chromosomes 21 into one of the daughter cells, the oocyte or the 1^st ^polar body rather than primary non-disjunction of two chromosomes 21 [2-4,11-14]. (cc Figures 2 and 4)

### The maternal age effect may be due to differential selection and accumulation of T21 oocytes in the ovarian reserve of older women

**Figure 5:**
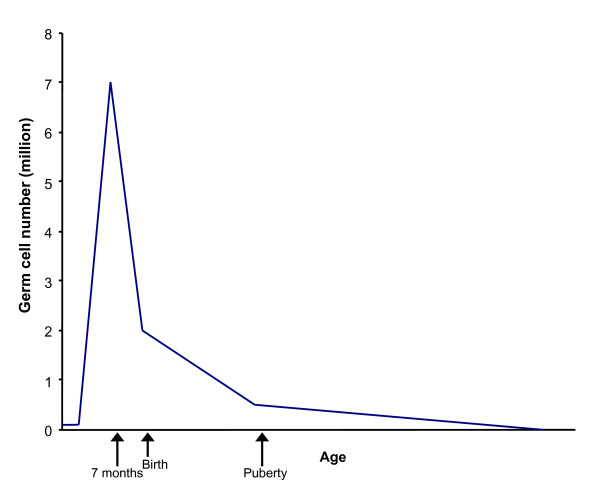
**Changes in human oocyte number during prenatal and postnatal development**. There is a very rapid increase in human female germ cell (oocyte) number early during fetal development with a peak at 7 months gestational age, followed by a relatively rapid decline before birth and postnatally before puberty, but a slower depletion during reproductive years until menopause

A second question is whether this type of ovarian mosaicism might also explain the maternal age effect (Figure 1). We suggest that it does. It has been demonstrated by analysis of chromosome behavior in cases of foetal T21 that there is a substantial delay in foetal oocyte maturation in comparison to that seen in cases with a normal karyotype [12-17]. It seems reasonable to conclude that T21 foetal oocytes may lag behind the normal during foetal development, when there is a dramatic reduction in numbers by apoptosis from age 20 weeks until birth and then postnatally until puberty (Figure 5). It is also possible that there is a further selection against T21 oocytes leading up to the total 300–400 maturing to ovulation between puberty and menopause as discussed with respect to the oocyte selection model proposed by Zeng and co-workers [18,19]. The net effect of this situation is that any T21 oocytes in the original pool will comprise a larger proportion of the ovarian reserve at later maternal ages.

### The increased recurrence in young women may be due to high grade T21 ovarian mosaicism

The third issue concerns the origin of the higher incidence in younger women having a second child with T21 DS. It is likely that there is a wider variation in ovarian T21 mosaicism among human females than is apparent in our relatively small sample. Thus some exceptional women may have a high proportion of T21 oocytes in their ovaries, which could explain the increased recurrence. This proposal is supported by previous reports on ovarian mosaicism in seven normal adult women who have had children with T21. Remarkably, in all these seven cases T21 cells were identified either in ovarian biopsy material or ovulated oocytes from these adult women (Table 2) [20-26].

**Table 2:** Previous studies of T21 mosaicism in mothers with Down's pregnancies where not only blood and skin but also ovarian cells have been analysed

No. of DS pregnancies	Percentage T21 Cells (%) Maternal Tissue Sample				Reference
		
	Blood	Skin	Ovary		
1	(6.0)*	(6.0)*	left	(92.0) *	Taylor et al. 1970
			right	(88.5) *	
1	2/101 (1.98)	0/92 (0.0)	2/35	(5.71)	Parke et al. 1980
1	12/162 (7.4)	82/102 (2.0)	left 22/100	(22.0)	Uchida et al 1985
			right 84/100	(84.0)	
9	0/260 (0.0)	0/21(0.0)	12/79	(15.2)	Nielsen et al 1988
4	3/100 (3.0)	(14.0)*	left	(47.0) *	Sachs et al. 1990
			right	(44.0) *	
3	(0.0)*		8/20	(0.4)	Tseng et al 1994
4	(0.0)*		3/4 oocytes	(75.0)	Cozzi et al 1999
			4/7 embryos	(57.0)	

*) No of analysed cells not specified

Note: In the Case of Cozzi et al 1999 embryos were also investigated

### Aberrant maternal chromosome 21 recombination detected by family linkage analysis is the same as that expected in T21 oocytes

An additional outstanding question concerns the most likely explanation for the aberrant maternal recombination previously detected by family linkage analysis [2-4]. On the basis of our experience in meiotic chromosome behavior of T21 oocytes in foetal ovaries we suggest that these recombination patterns are precisely those expected from the pairing, crossing-over and segregation of the three homologs 21 (Figure 4) [12-16]. One alternative is the formation of a bivalent plus a univalent, where the bivalent will show normal crossing-over, chiasma formation and recombination, while the two half chromosomes (chromatids) of the (achiasmatic) univalent may segregate in the same way as during mitosis, this behavior also explaining the common occurrence of extra chromatids in unfertilized oocytes at the metaphase II stage investigated at Assisted Conception Units [12,27]. Another alternative is the formation of a trivalent, where pairing problems including stalling is likely to lead to altered recombination in proximal and/or distal positions [16,28].

### The low paternal origin of T21 may be due to more effective selection against T21 germ cells during spermatogenesis than oogenesis

We may in addition ask what the underlying reason might be for the relatively low frequency (around 10%) of T21 DS, where the extra chromosome 21 is paternally inherited [2-4]. One explanation could be the existence of similar degrees of mosaicism in foetal testes, but a more efficient selection against aberrant cells during spermatogenesis than oogenesis [29-31]. An indication that some T21 spermatocytes may nevertheless progress to reach first meiotic metaphase in the mature testes originates from studies of DS males post puberty [32-35]. These cases are characterized by substantially reduced numbers of spermatocytes reaching the first meiotic metaphase. Interestingly, the three chromosomes 21 then show the same principal pairing, crossing-over, chiasma formation and recombination abnormality as described above for T21 oocytes, which also concords with that expected from Drosophila and mouse experimentation [36-38]. Two types of T21 spermatocytes are seen at the metaphase I stage, either containing a bivalent plus a univalent 21 or a trivalent 21. Trivalents show chiasmata in aberrant positions, and univalents are expected to be liable to precocious anaphase I separation, leading to extra or missing chromatids in daughter cells (Figure 4).

### Parental aneuploidy gonadal mosaicism may be the major underlying reason for T21 conceptions

In summary, we suggest that gonadal T21 mosaicism may be a prevailing constitution in humans, underlying the different types of predisposition for having a child with T21 DS. We presume that for any particular parent this is dependent on the proportion of T21 cells in their adult gonads resulting from the differential delay and selection during oogenesis and spermatogenesis. Thus some women, who are high grade ovarian mosaics, are predisposed to T21 offspring at an early age, but for the low grade majority the delay in T21 oocyte maturation leads to their accumulation within the ovarian reserve and a higher incidence at later reproductive ages. On the other hand, the more efficient apoptotic selection against aneuploid germ cells in testes implies that men are less likely to father a child with DS, although exceptions to this general rule are known as regards some who are high grade testicular mosaics, the first such cases identified already in 1971 [39].

### Parental gonadal aneuploidy mosaicism may be the major reason for the high incidence of aneuploid conceptions in the human population

We further presume that the existence of gonadal mosaicism as regards T21 may only be the tip of the iceberg, and this mechanism may be more general. It seems likely that gonadal mosaicism is the main causative factor not only for the origin of T21 but also for other aneuploidy conditions, where only a few are compatible with postnatal life, i.e. common numerical sex chromosome abnormalities such as XXY Klinefelter, XYY, XXX as well as the more rare X Turner together with trisomies 13 Patau and 18 Edwards syndromes.

Finally, it is important to note that there are a number of previous reports in the literature that indicate the occurrence of parental gonadal mosaicism as the causative factor, although this mechanism is generally thought to be quite rare [40,41]. Further studies will be required to find out if our model on gonadal mosaicism leading to secondary meiotic non-disjunction constitutes the only source of origin of aneuploidy conceptions in the human population, or if other mechanisms might also contribute to this effect [2-4,41].

## Conclusion

In this study we have, for the first time, documented that T21 ovarian mosaicism is common in normal human female foetuses. T21 mosaicism has also previously been documented in ovaries from adult human females, who have had one or more children with T21 DS (Table 1). Thus, in a total population of 15 human females, where ovarian cells have been investigated in this respect, T21 mosaicism of varying degrees has been found in all. On the basis of these observations, together with the expected implications as regards the maternal age effect, the higher recurrence of DS in younger women, the aberrant maternal meiotic recombination patterns and the low incidence of DS of paternal origin, we suggest that gonadal mosaicism is a prevalent unifying reason for T21 conceptions in the human population.

## Perspectives

In this paper we challenge the current dogma that disomic maternal and paternal gametes are most often caused by failure of the two homologs 21 to separate at the first meiotic division, so called primary non-disjunction. Instead we propose that obligatory secondary non-disjunction of three homologs 21, which is the expected outcome of gonadal T21 mosaicism, may constitute a common reason. One relatively straight forward way to obtain further information on this situation would be to count copy number of chromosome 21 at the meiotic metaphase I stage to find out what proportion contain two respectively three copies. To our knowledge no T21 spermatocytes have so far been identified at the metaphase 1 stage in testicular biopsy samples from any men other than those diagnosed as having T21 DS, but this nevertheless requires further study. In particular, there are as far as we are aware no such data available on human oocytes, where collection of a large enough sample of ovulating oocytes presents one of the outstanding hurdles. Further studies in this regard are underway. Obviously more work is also required to find out to what extent gonadal mosaicism does exist for other chromosomes than 21, including those that are of special relevance for common genetic disease in the human population. On another note, we are also wondering if the intriguing species difference as regards constitutional aneuploidy, where it would appear that our own species is much more affected than other mammals, could be caused by more stringent control of embryonic cells divisions and therefore a lower incidence of gonadal aneuploidy mosaicism. Similar studies as we have here reported on chromosome copy number in human foetal ovaries should be straight forward and allow this outstanding question to be readily answered.

## Competing interests

The authors declare that they have no competing interests.

## Authors' contributions

MH designed the study, obtained the funding from BBSRC and the local ethical approval, obtained Case samples 1–4, supervised the practical work and wrote the initial draft of the paper; SP, MT and EI performed the immunofluorescence and FISH analysis; MW, NP and AMJ obtained local ethical approval, obtained Case samples 5–8 and together with EI obtained the funding from The Swedish Research Council and Stockholm City Council; All the authors contributed to and have approved the final version of the manuscript.
